# Solar-mediated thermo-electrochemical oxidation of sodium dodecyl benzene sulfonate by modulating the effective oxidation potential and pathway for green remediation of wastewater

**DOI:** 10.1038/srep44683

**Published:** 2017-03-15

**Authors:** Di Gu, Simeng Gao, TingTing Jiang, Baohui Wang

**Affiliations:** 1 Institute of New Energy Chemistry and Environmental Science, College of Chemistry and Chemical Engineering, Northeast Petroleum University, Daqing 163318, PR China

## Abstract

To match the relentless pursuit of three research hot points - efficient solar utilization, green and sustainable remediation of wastewater and advanced oxidation processes, solar-mediated thermo-electrochemical oxidation of surfactant was proposed and developed for green remediation of surfactant wastewater. The solar thermal electrochemical process (STEP), fully driven with solar energy to electric energy and heat and without an input of other energy, sustainably serves as efficient thermo-electrochemical oxidation of surfactant, exemplified by SDBS, in wastewater with the synergistic production of hydrogen. The electrooxidation-resistant surfactant is thermo-electrochemically oxidized to CO_2_ while hydrogen gas is generated by lowing effective oxidation potential and suppressing the oxidation activation energy originated from the combination of thermochemical and electrochemical effect. A clear conclusion on the mechanism of SDBS degradation can be proposed and discussed based on the theoretical analysis of electrochemical potential by quantum chemical method and experimental analysis of the CV, TG, GC, FT-IR, UV-vis, Fluorescence spectra and TOC. The degradation data provide a pilot for the treatment of SDBS wastewater that appears to occur via desulfonation followed by aromatic-ring opening. The solar thermal utilization that can initiate the desulfonation and activation of SDBS becomes one key step in the degradation process.

Surfactants are widely utilized in many applications such as metal processing, textile, food, pharmaceuticals and paper industries^1^. Due to the biotoxicity and non-biodegradability^2^, wastewater containing surfactants need to be treated before discharging into the aquatic environment, in terms of public and environmental health. For the treatment of the surfactant wastewater, many advanced technologies have been applied by focusing on physical methods^3^,^4^,^5^ such as foam fractionation technology, adsorption method and membrane separation technology; chemical methods^6^,^7^,^8^,^9^,^10^,^11^ such as flocculation precipitation method, coagulation sedimentation method, advanced oxidation processes (AOPs) by photocatalytic degradation and photoassisted fenton oxidation; biological methods^12^,^13^,^14^ such as the natural degradation and biodegradation, and combination of above methods^15^,^16^.

Whereas, to date no satisfactory methods have been developed to the specific and efficient degradation of surfactant in wastewater. All the above-mentioned methods require several pretreatment steps depending of the nature of the wastewater and the methodology. The pretreatment should guarantee a minimum loss of energy, no or minimum contamination, complete decomposition of organic matter and a minimum number of steps. Also, it should be economical (e.g. no or low consumption of chemicals) and environmentally friendly (“green remediation”). Therefore, the development of new technologies that can be able effectively and environment-kindly to mineralize surfactants has become an urgent challenge.

The STEP (Solar thermal electrochemical process) has been demonstrated successfully by driving chemical reaction by using solar thermo- and electrochemistry to minimize the fossil energy, meanwhile, maximize the rate of electrolysis reactions in our past studies^17^,^18^,^19^,^20^,^21^,^22^. Sodium dodecyl benzene sulfonate (SDBS) has been widely used in several industries such as food, textile, paints, pesticides, pharmaceutical, oil recovery, and paper^23^,^24^,^25^,^26^. It’s the major components of laundry detergent, bar soaps and so on^23^. The data in the experimental section demonstrate that SDBS were identified and targeted in three kinds of wastewater even though the treated wastewater from the domestic sewage treatment plant.

The immune-toxicity of SDBS has been found that its immunity function was inhibited while the exposure concentration was higher than 0.40 mg/L^27^. Therefore, seeking out an effective way to deal with SDBS wastewater becomes urgent and important. In the present work, the STEP process was extended and adapted to a study of the efficient treatment of the surfactant wastewater, exemplified by popular SDBS wastewater. The goal is that the green remediation makes tough SDBS easily and fully mineralize into CO_2_ and H_2_O.

SDBS consists of a benzene ring coupled with a large aliphatic chain and a sulfonic group (Fig. S1, ESI). By the calculation of density functional theory at the B3LYP/6-31G(d) level, the degradation potential of SDBS can be found to decrease with the increasing of temperature in this paper. Further, the experimental degradation showed that, compared with the conventional electrochemical methods with the degradation potential in room temperature (E_RT_) of 20 V^28^, high efficiency and fast degradation rate were displayed in lower potential with STEP mode (E_T_) due to the sulfonate was removed and the SDBS got activation under the thermal effect. The SDBS, toughly electrooxidized in the conventional method, is thermo-electrochemically oxidized to CO_2_ at the anode while H_2_ is generated at the cathode by lowing effective oxidation potential which is less than the electrolytic potential of water and suppressing the oxidation activation energy which originated from the combination of thermochemical and electrochemical effect. The SDBS was oxidized and decomposed rapidly after electrolysis in high temperature with the adaptation and adoption of solar STEP concept via desulfonation followed by aromatic-ring opening, and the solar heat that can initiate the desulfonation and activation of SDBS become one key factor. A perfect, energy-free, sustainable wastewater treatment is smartly established by accomplishing the task of efficient solar utilization, green and sustainable remediation of wastewater and advanced oxidation processes.

## Results and Discussion

### Thermodynamics and Potential of Thermo-dependent Electrochemical Degradation

**Figure 1:**
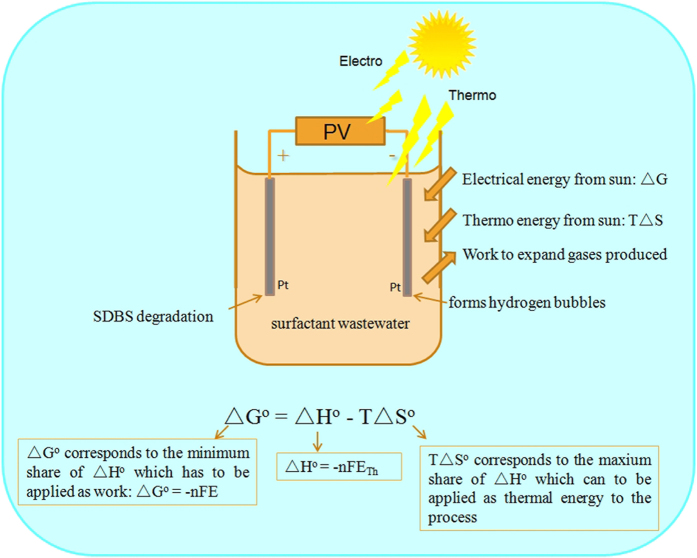
Thermodynamics of SDBS degradation (PV is photovoltaic system).

Solar-mediated thermo-electrochemical oxidation of SDBS is the decomposition of SDBS into CO_2_ and H_2_ due to current being passed through the wastewater. The minimum voltage of electrolytic cell to drive SDBS electrolysis is the reversible (no losses in the process) potential as shown in Fig. 1:


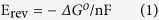


Here, n = number of electron transferred, F = Faraday’s constant (F = 96485 C/mol). Thermo corresponding to TΔS can be integrated into the process. The potential at which the cell operates adiabatically (heat is not lost or required) is the thermoneutral potential:


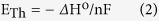


E_Th_ accounts for the “additive energy” when thermal energy be added from the solar energy (high temperature process). E_Th_ is lower than E_rev_ as it contains the heat associated with the entropy change for the reaction. This provides an opportunity to comprehensive utilize the thermo energy and electrical energy in solar energy that is inevitably produced, in this way, the overall electricity consumption and, thereby, the wastewater treatment and H_2_ production price can be reduced.

#### Electrode Reaction of SDBS Treatment by STEP

The STEP chemistry is concerned with the two aspects of the solar utilization related to the improvement of the conversion efficiency, and the chemical reaction in view of the change of thermodynamic and kinetic basis. For the aspects of chemical reaction, it must be the pursuit of efficiency and selectivity, and these two points will be completely determined by the nature of the reaction. According to the characteristics of the SDBS oxidation itself, the STEP chemical process is supposed to be determined by the thermodynamics and kinetics in combination of the efficient solar conversion and effective chemical reaction. The electrochemical oxidation of SDBS can occur directly through electron transfer between the anode and SDBS. Eqs 3, 4, 5 summarize the electrode reaction of SDBS treatment by STEP, electrochemical oxidation of SDBS to carbon dioxide in anode (Eq. 3) and hydrogen evolution in cathode (Eq. 4),


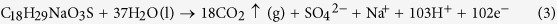



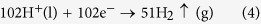


Full cell reaction:


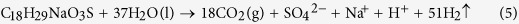


Sunlight can drive electrochemical oxidation with the release cathodic hydrogen. In the process of electrochemical oxidation of SDBS wastewater, the electrolytic decomposition of the water leads to the simultaneous generation of a co-product H_2_, which is useful as a fuel.

#### Thermodyanmic calculation of the electrochemical potential by DFT

For description of the effect of elevator of temperature on the process of electrolytic reaction, Nernst equation is used to illustrate the relationship between the electrolytic potential and free-energy (G) which are critical thermodynamic properties of a compound allowing a chemist to determine the energy changes of a chemical reaction. Since the thermodynamic data of SDBS involved in the processes is not available neither in the NIST Webbook (WB) nor Computational Chemistry Comparison and Benchmark Database (CC). A quantum chemical method were performed to investigate temperature effect on free energies for the full cell reaction.

We have employed the density functional theoretical (DFT) methods at the B3LYP/6-31 G(d) level to understand the free energy of the gas reaction above. The geometries and zero-point energies (scaled by 0.9806) were calculated at the B3LYP/6-31 G(d) level of theory. Thermal corrections were assessed at 298 K, 303 K, 323 K, 343 K and 363 K to investigated temperature effects on the free energies of reaction. The gas phase free energy changes from 298k to 363k are shown in Table S1 (ESI). The calculations indicate that △ G of the reaction is declined with the increasing of temperature. The electrolysis potentials are then calculated from the thermodynamic free energy components of the reactants and products. At unit activity and at any electrolysis temperature, T_STEP_, the reaction has electrochemical potential, E°_T_.


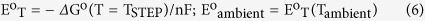


where E°_T_ is the standard-state cell voltage, T_ambient_ = 298.15 K

**Figure 2:**
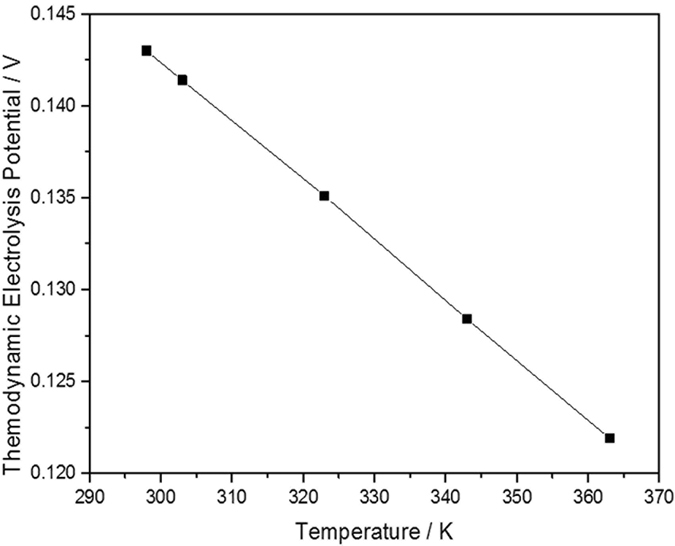
Thermodynamic calculation of the electrochemical potential of the electrochemical potential of SDBS oxidation using B3LYP/6-31 G(d) level of theory.

According to E_T = _-E°_T_, electrolytic cell reaction to obtain potential. The variation of the SDBS electrolysis potential with temperature is calculated and presented in Fig. 2. The SDBS oxidation potential decreases with the increasing of temperature, which means that the STEP process can be applied to SDBS electro-oxidation. For example, from 298 K to 363 K, the electrolysis potential in the aqueous phase decreases from 0.1430 V to 0.1219 V. This decrease provides a theoretical basis of the STEP process for effective and efficient removal of SDBS from wastewater.

As calculated in thermodynamics, the electrochemical driving force (potential) for a variety of chemicals will be shown to significantly decrease with increasing temperature. The oxidation potentials decrease more rapidly with increasing temperature, indicating that the STEP electrochemical energy may be readily and fully applied to electrooxidation of surfactant wastewater without the water splitting by and pathway. The STEP process of SDBS degradation can be conducted by modulating the effective oxidation potential and elevation of the input port of the solar heat for lifting the overall solar utilization.

### STEP Treatment of SDBS

#### Thermo-dependent CV Measurement of SDBS

**Figure 3:**
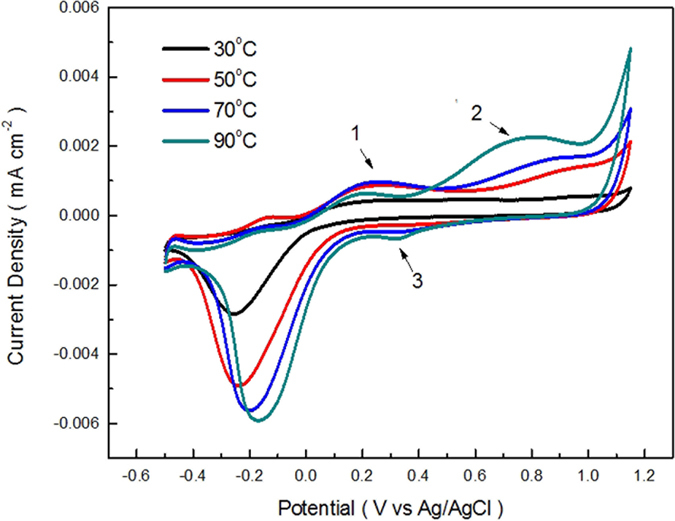
Thermo-dependent CV of SDBS aqueous solution with the changing of temperature.

The Fig. 3 presents the thermo-dependent cyclic voltammogram of SDBS aqueous in varied temperatures. There are two anodic peaks of SDBS oxidation appear in the temperatures about 50 °C, at a potential range toward a positive scanning, less than the oxygen evolution potential. Under the condition of high temperature electrolysis, H + as electrophilic reagents can directly attack benzene ring to produce alkylbenzene via electrophilic substitution reaction corresponding to peak 1. Desulfonation mechanism have been proved that it can be more easily with the increasing of temperature under the conditions of H^+^ coexistence, therefore, part of SDBS has converted into alkylbenzene when the temperature goes up to 90 °C before the addition of electric field, corresponding to the peak 1 of 90 °C is lower than 50 °C and 70 °C. It favors the further electrolysis of alkylbenzene to intermediates, corresponding to the peak 2, in order to reach the aim of saving electric energy and efficient use of full spectrum solar energy.

Peaks 2 of 30 °C and 50 °C in Fig. 3 applies that the peak intensity of anodic oxidation, which even can not have a clear distinction between the peak of oxygen evolution, raised by the enhancement of temperature, simultaneously, the anodic oxidation peak potential decrease which was detailed in Table 1. The decrease in peak oxidation potential from 1.00876 to 0.79392 V (vs. Ag/AgCl) is consistent with the synergy of a thermodynamic potential drop with the temperature increase, combined with a kinetic overpotential drop expected with facilitated electron transfer at higher temperatures. The results are conducive to the inclusion of solar thermal energy to improve the degradation efficiency and decrease the energy for the STEP degradation of SDBS wastewater.

#### Effect of the controlled potential on the oxidation reactions

**Figure 4:**
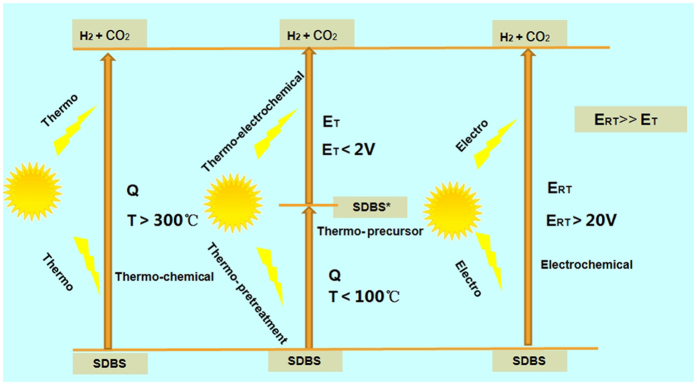
Model of STEP degradation of SDBS in wastewater.

Figure 4 provides a clear understanding about the improvement in SDBS oxidation with the increasing of solar heating, and the SDBS electrolysis potential decreases with the increasing of temperature. For a case, both main reaction and side reaction occur in the oxidation of SDBS in the same time:

SDBS → CO_2_ (Oxidation of SDBS, main reaction)

Water → O_2_ + H_2_ (Water splitting, side reaction)

The *E*_*SDBS-oxidation*_ overlapping or more than *E*_*water-splitting*_ can be encountered in the majority of reactions. The bypassing current will arise due to the water splitting. As a result, the electrochemical (coulombic) efficiency is significantly decreased. So, on the basis of the thermodynamics, the remarkable shift will be subjected to an adjustment of the potential for a suitable separation of *E*_*SDBS-oxidation*_ and *E*_*water-splitting*_, which is shown obviously in Fig. 3 under the temperature of 70 °C and 90 °C.

#### Effect of the Temperature on the Oxidation Reactions

**Figure 5:**
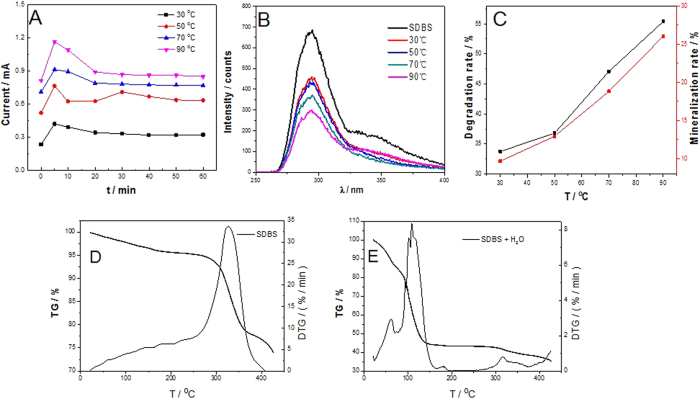
(**A**) I-t curve of STEP electrolysis SDBS in NaCl as the electrolyte at different temperatures; (**B**) The fluorescence spectra of SDBS in different temperature after 60 min; (**C**) The degradation rate of SDBS by the photo absorption and mineralization of SDBS by TOC monitoring with the increasing of temperature after 60 min oxidation reactions. TG and DTG curves of SDBS power (**D**) and SDBS cream (mixed with water) (**E**).

In order to confirm the improvement of temperature in efficiency resulting from the use of the STEP procedure, I-t curve of STEP electrolysis in the presence of the different temperature and subsequent degradation rate of SDBS was checked. Figure 5(A) shows the I-t curve of SDBS electrolysis with 1.2 V which is under the electrolytic voltage of water, avoiding the interference of water electrolysis, illustrating that the current increases with the increasing of temperature. It can be concluded from the curve that the solar heat is conducive to the free ion movement in the solution to have a higher current, which could induce a higher degradation rate of SDBS.

There is the conjugate system which can produce fluorescence in SDBS molecules. Figure 5B is SDBS fluorescence spectra of SDBS in different temperature after 60 min. It can be seen from the figure that there is no definite increase of degradation rate from (peak height of λ = 290 nm) 30 °C to 50 °C, compared with the obviously increase of degradation degree from 50 °C to 70 °C and from 70 °C to 90 °C. The growing tendency is concluded in Fig. 5C (black line), which have a clear impression on the acceleration of degradation reaction responded from the slope of the curve. The degradation rate in Fig. 5C reflected that the vanish of SDBS, which could break down to organic intermediates or to CO_2_ directly. The mineralization rate of SDBS (Fig. 5C, red line), which was monitored by TOC with the increasing of temperature, was used to indicate that the organic substance directly and completely degraded to CO_2_ and H_2_O. The mineralization rate is enhanced with the degradation rate of SDBS, indicating that the STEP process can remove SDBS in wastewater efficiently with the increasing of temperature and save energy consumption.

The influence of thermal on the degradation was investigated through TG and DTG curves in Fig. 5(D) and (E). The DTG curve in Fig. 5E has a small absorbing peak at 60 °C, indicating that the reaction of desulfonation have been proved. It can be known from figure that for the thermochemical efficiency of the STEP degradation reaction of SDBS, thermochemical effect was combine with the desulfonation of SDBS to conduct a changing process. It is related to the heat energy associated with chemical reactions and/or physical transformations including thermoactivation and thermochemical reaction.

For dominated factors and simplified conditions, the actual wastewaters containing SDBS and commercial detergent were sampled. It can be concluded from the Table 2 that the STEP mode can effective improve the degradation rate of the surfactant in the real wastewater. It is clear to obtain a great advantage in the combination of solar-thermo and solar-electro. The solar thermo-energy account for more than four percent of the solar energy and the conversion of the efficiency of absorption of solar thermal energy is 65–80%^17^. Hence, if as many as solar thermal energy can be used in a reaction and with high efficiency, it is a cost-effective way of utilization of solar energy in wastewater treatment.

### Product Analysis and Mechanism of SDBS Degradation

#### Product Analysis

**Figure 6:**
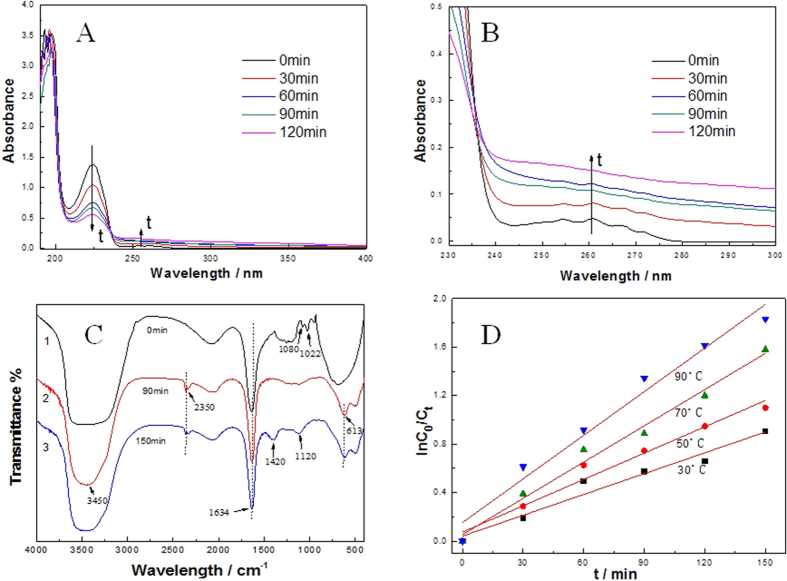
(**A** and **B**) UV-vis absorption spectroscopy of the SDBS aqueous solution at 50 °C; (**C**) FT-IR spectra of SDBS aqueous solution(1), after 60 min degradation(2) and 120 min degradation at 50 °C (3); (**D**) The first-order kinetics of SDBS degradation.

The changes in UV–vis absorbance characteristics of SDBS were investigated from 200 to 400 nm during the electrochemical degradation process and the results are shown in Fig. 6A and B. It can be seen that a maximum absorbance peak at 224 nm which represents the SDBS and disappears gradually during the electrochemical oxidation process. It can be concluded that the concentration of SDBS decreases when peak height of 224 nm was lower and the concentration of intermediates increase when the peak heights of greater than 240 nm were higher.

To track the degradation behaviors of SDBS in wastewater, the FT-IR spectra probed at different reaction time was carried out between 4000 and 400 cm^−1^, as shown in Fig. 6C. Despite some of small absorption peaks were changed after 120 minutes of degradation, the characteristic absorption of benzene ring (1634 cm^−1^) was still exist, corresponding that the part degradation of SDBS. Figures 6C–2 showed the obvious characteristic absorption peaks of SO_4_^2-^ in 613 cm^−1^, meanwhile, the obvious characteristic absorption peaks of -SO_3_ in 1022 cm^−1^ disappeared, which meant the sulfonic group was oxidized to sulfate under STEP degradation for 60 min. Furthermore, a new peak in 2350 cm^−1^ was appeared, which can be assigned to the formation of HCO_3_^−^, illustrating that part of the linear alkyl and benzene was degraded. As time adds to the STEP degradation, the characteristic absorption peaks of SO_4_^2−^ in 1120 cm^−1^ was appeared, illustrating that the mount of SO_4_^2−^ was increasing. Alongside a new peak in 1420 cm^−1^ was appeared, which can be assigned to the formation of CO_3_^2−^, illustrating that the linear alkyl and benzene was further removed and the formation of CO_2_. In addition, the wave number is located at 3450 cm^−1^ for the -OH (hydroxyl) characteristic absorption peak, mainly due to the hydrogen bond caused by the water in the water sample. Together these provide evidence that the principal product of the degradation of SDBS is carbon dioxide.

Gas chromatographic analysis of the composition of gas from SDBS degradation is shown in Table 3. The formation of CO_2_ proved that the alkylbenzene was completely degraded to CO_2_ and H_2_ in STEP. In addition, we can see that, with the increase of temperature, the amount of CO_2_ was gradually increased. Using CO_2_ as the target product, the current efficiency of SDBS degradation in 90 °C almost two times as many as 30 °C, further explains the theory of STEP high temperature is more conducive to the degradation of surfactant wastewater.

#### Kinetics of SDBS Degradation

The reaction order is the basic parameter of chemical kinetics. It is clearly observed in Fig. 6D and Table S2 (ESI) that the kinetics keeps one order from the stage of 30 °C to 90 °C. The same mechanism of the reaction must be followed The rate is increased to k_90°C_/k_30°C_ = 2 times by an increment of 60 °C heat. The event obeys the Arrhenius equation of temperature-dependence. In the present work, the STEP degradation of SDBS is investigated with emphasis on the effect of several operating parameters as well as various matrix components on SDBS degradation kinetics.

#### Mechanism and pathway

In reaction, the SDBS oxidation by: (i) direct electron transfer to the anode surface, (ii) heterogeneous reactive oxygen species produced as intermediates of oxidation of water to oxygen, including the powerful physisorbed •OH at the anode surface, generated via Eq. 7, and weaker oxidants like H_2_O_2_ produced from •OH dimerization by Eq. 8 and O_3_ formed from water discharge at the anode surface by Eq. 9/or (iii) other weaker oxidant agents electrochemically produced from ions existing in the bulk^29^,^30^.


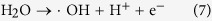



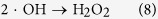



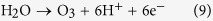


Furthermore, the electrooxidation process can be improved by the action of oxidants like active chlorine species, persulfate, perphosphate, percarbonate and H_2_O_2_ that are electrochemically generated from agents existing in the bulk solution such as chloride, sulfate, phosphate, carbonate and oxygen, respectively^31^. We use the sodium chloride as the electrolyte in our STEP reaction. Active chlorine species are formed and as the main indirect oxidation agents employed in wastewater treatment. The oxidation with active chlorine is based on the following Eqs^32^.


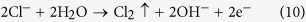



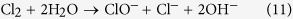



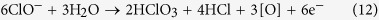



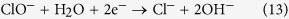


**Figure 7:**
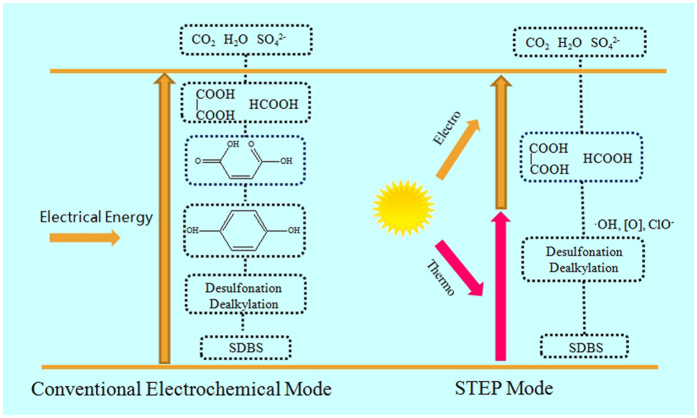
Mode of Conventional Electrochemical and STEP degradation of SDBS.

The kinetic energy (the kinetic energy and the rate are positively correlated) increases with the increasing of temperature. When the kinetic energy of the water molecule reaches or exceeds the bond energy of covalent bond, the molecule may dissociate into free radical. So the higher the temperature, the more molecules that dissociate into free radicals with strong oxidizing property^33^. When SDBS is attacked by massive free radicals, the intermediates are degraded to carboxylic acid quickly in the STEP Mode (Fig. 7). Accordingly, combined effect of strong oxidizing of these products, accelerated the degradation rate of SDBS in wastewater. A conclusion on the mechanism of SDBS degradation by STEP can be schematically proposed in Fig. 7 based the analysis of the CV, TG, GC, FT-IR, UV-vis and fluorescence spectra.

The STEP degradation of SDBS in wastewater opens up an alternative “green” degradation route, which is surely efficient and without carbon footprint. The kinetic analysis illustrated that large numbers of free radicals in the STEP mode (Fig. 7) can effectively speed up the reaction process, making the intermediates rapidly break down to small molecular organic compounds and then CO_2_.

Compared with the toxic SDBS to aquatic organisms, products of the full mineralization such as CO_2_ and H_2_O are non-toxic, and the degraded intermediates in STEP mode such as short life of organic acids, etc. are less toxic 34,35,36, which are easily destroyed and disappear in the echo system. Some data of the LD50 (half lethal dose) were searched as a demonstration of toxicity by pollutant. The LD 50 of SDBS is 2.3 mg/ kg^37^ and mineralized products of CO_2_ and H_2_O are non. The dominated intermediates: acetic acid with the LD50 3.31 g/kg^38^, formic acid with the LD50 1.1 g/kg^39^, and other intermediates with the LD50 much more larger than SDBS. Toxicity of the wastewater decreased obviously during the STEP mode with the increasing of LD50 index after treatment. Solar thermal provides energy to increase the degradation rate of SDBS in wastewater, meanwhile, a novel and energy-free route for wastewater treatment accomplished by the synergistic use of solar energy.

### Summary and perspective

**Figure 8:**
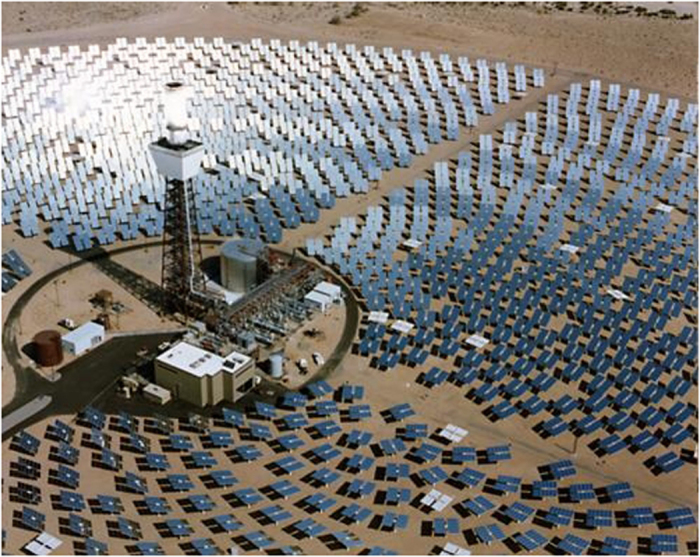
Solar power tower in Daggett, California (From Wikipedia).

Figure 8 is the picture of a 10-MW solar thermal electric power plant. Heliostats followed the sun and directed its light to the receiver at the top of the tower. It is one of several energy technology start-ups pursuing concentrating solar power, where lenses and mirrors concentrate sunlight to make heat.

**Figure 9:**
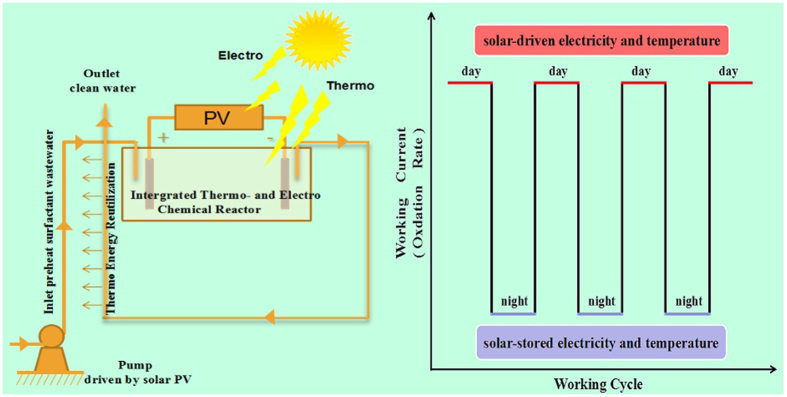
Robotic work for treatment of surfactant wastewater tuned by solar energy without input of any energy and management.

The robotic operation for treatment of surfactant wastewater for industrialization via the successful application of our studies combined with solar power tower can work around the clock in Fig. 9. The system is directly driven by solar in the daytime, and operated by the stored battery and heat in the dark time. The working condition will be estimated like the curve shown in the Fig. 9. During the daytime, the temperature can maintain the temperature at 90 °C (or above the point), which can gain a high current (fast oxidation). On the night shift, there is no solar, but the stored electricity and heat play an important role for preserving an appropriate current of the oxidation. In summary, the STEP-surfactant oxidation process can be a continuous process, which is fully driven by solar energy without the input of any other forms of energy.

## Conclusions

This paper presents the first application of STEP concept to degrade SDBS in wastewater. This new approach degrade surfactant pollutants to hydrogen combines solar thermal energy and electrolysis, to open a new, facile route to surfactant wastewater treatment and simultaneous hydrogen production. And the switch of the anodic oxidation of water to organic pollutants toward CO_2_ evolution instead of O_2_ evolution decreases the anode potential with greater electrochemical efficiency by compared with solely water-splitting to hydrogen. The electrolysis of SDBS appears to occur via desulfonation followed by aromatic-ring opening, and the solar thermal that can initiates the desulfonation and activation of SDBS in the reaction becoming the key factor in the degradation process. The degradation of SDBS was fully driven by solar energy without the input of any other forms of energy and chemical additive, with the increasing of temperature, there is a decrease in the requisite calculated and observed degradation potential. The rate of SDBS degradation are significantly enhanced by solar thermal heating, and the formation of reduction product is inhibited, and as evidenced by the significant increase of yield at 90 °C compared to 30 °C.

By using this solar thermal electrochemical process, we have demonstrated a new method for the surfactant wastewater treatment to hydrogen production with higher efficiency than either electrochemical or photovoltaic conversion process acting alone. The theoretical and experimental ease of the STEP process presents evidence that degradation of pollutants plus hydrogen production will be applicable to a broad range of STEP process. Simply, the merits of the STEP are dominated by the full utilization of solar energy for a sustainability, substantial decrease of the redox potential for enhancement of electrochemical efficiency, effective elevation of the input port of the solar heat for lifting the overall solar utilization. The three-field from solar energy (solar photo-, solar electro-, and solar thermo-), for high efficient SDBS degradation at the TiO_2_ nanotubes electrode by adjusting the three-field will be investigated in an expanded study in the future.

## Experimental Section

### Chemicals and Materials

The SDBS wastewater was prepared with deionized water. SDBS(C_18_H_29_NaO_3_S, 99.0%), sodium sulphate (Na_2_SO_4_, AR) and sodium chloride (NaCl, AR) were purchased in DM tianjing reagent Co., Ltd. and used as received.

### Analysis of actual SDBS wastewaters

Three kinds of actual wastewaters containing SDBS were sampled and pretreated from the discharged wastewater of a domestic sewage treatment plant, fluent wastewater of production sector of an alkylbenzene sulfonates plant and washing wastewater of labwares in the Daqing region. The analysis of the compositions was performed by the conventional chemical and instrumental methods. For dominated factors and simplified conditions, the third kind of the wastewater was selected as the actual wastewaters in our experiment. One sample containing SDBS as main component was sampled as “Wastewater 1”, and another commercial detergent (Diaopai, China) was sampled as “Wastewater 2”. Both the samples were prepared by tap water.

Main data are listed as SDBS 26 mg/L, COD 87 mg/L (domestic sewage treatment plant), SDBS 80 mg/L, COD 210 mg/L (alkylbenzene sulfonates plant), SDBS 50 mg/L, COD 130 mg/L (Wastewater 1, main contribution is SDBS).

### Thermo-dependent Cyclic Voltammetry

Thermo-dependent cyclic voltammetry (CV) experiments were performed with BAS Epsilon-EC electrochemical workstation at a sweep rate of 50 mV•s^−1^. A conventional three-electrode system was used, with both Pt sheets (20 mm × 20 mm) as working electrode and counter electrode, and Ag/AgCl as reference electrode. The cyclic voltammetry were performed with a high concentration of SDBS (1000 mg/L) to distinguish the peaks in sodium sulfate solution (Na_2_SO_4_, 5 g·L^−1^) as the electrolyte with the changing of temperature.

### Thermo-dependent Electrochemical Degradation

The experimental apparatus, described as our paper^21^ and detailed in electronic supporting information (ESI), combined with three parties of photoelectric, photothermal and electrochemical units (Thermo-Electro-Reactor) to treat the wastewater containing SDBS as shown in Fig. S2 (ESI).

Pt sheet (20 mm × 20 mm) working electrode and stainless steel sheet counter electrode were used in the SDBS wastewater degradation experiment. The concentration of SDBS is 50 mg/L in sodium chloride (NaCl, 5 g·L^−1^) as the electrolyte. A pH was adjusted to 7.0 prior to the experiment, via the addition of hydrochloric acid (HCl) and sodium hydroxide (NaOH).

### Product Analysis

The products of STEP degradation were identified by UV-Vis and FT-IR and the determined quantitatively by Gas Chromatograph. IR-spectra were measured in KBr pellets from 4000–400 cm^−1^ using a Tensor 27 FT-IR Spectrometer. UV-Vis Spectra samples were measured using a 1 cm path length quartz cell at room temperature with a UV-1700 Shimadzu Spectrophotometer using Cary Win UV Scan software. After 60 min with the current density 2.5 mA·cm^−2^, gas products were collected and analyzed using a gas chromatograph (GC-14C Shimadzu) installed with a flame ionization detector and a 30 m × 0.25 mm × 0.33 μm FFAP capillary column. The degradation rates of SDBS at different temperatures were determined using fluorescence spectrometry at a sweep rate of 50 nm•min^−1^ (LS-55, Perkin Elmer). Total organic carbon (TOC) was determined with TOC-LCPH/CPN (Shimadzu, Japan).

## Additional Information

**How to cite this article:** Gu, D. *et al*. Solar-mediated thermo-electrochemical oxidation of sodium dodecyl benzene sulfonate by modulating the effective oxidation potential and pathway for green remediation of wastewater. *Sci. Rep.* **7**, 44683; doi: 10.1038/srep44683 (2017).

**Publisher's note:** Springer Nature remains neutral with regard to jurisdictional claims in published maps and institutional affiliations.

## Supplementary Material

Supporting Information

## Figures and Tables

**Table 1:** Cyclic voltammetry of SDBS at varied temperatures (peak 2).

Temperature (°C)	Anodic peak potential.*V*_OxidationPeak_ (V vs Ag/AgCl)	Anodic peak current *J*_Oxidation Peak_ [μA/cm^2^]
30	1.00876	0.56
50	0.98923	1.43
70	0.93796	1.69
90	0.79392	2.26

**Table 2:** Degradation of the surfactant from real wastewater in 1.2 V.

Wastewater	Temperature (°C)	Degradation rate (%, 60 min)	Degradation rate (%, 180 min)	Mineralization rate (%, 180 min)
1	30	8.9	20.1	5.6
	90	28.2	62.3	33.2
2	30	7.9	19.6	7.3
	90	38.6	85.7	50.5

**Table 3:** Gas chromatographic analysis of the composition of gas from SDBS degradation in different temperature after 60 min.

Temperature(°C)	Gas product (mL)	
	H_2_	CO_2_
30	0.282	0.0144
50	0.414	0.0175
70	0.607	0.0199
90	0.794	0.0220
