# Supplementary material for: Solar-mediated thermo-electrochemical oxidation of sodium dodecyl benzene sulfonate by modulating the effective oxidation potential and pathway for green remediation of wastewater
Source: Sci Rep. 2017 Mar 15;7:44683. doi: 10.1038/srep44683 (PMC5353698; doi:10.1038/srep44683)
Supplement: Supporting Information [file srep44683-s1.doc]

**Solar-mediated thermo-electrochemical oxidation of sodium dodecyl benzene sulfonate by modulating the effective oxidation potential and pathway for green remediation of wastewater**

**Di Gu, Simeng Gao, TingTing Jiang and Baohui Wang^*^**

Institute of New Energy Chemistry and Environmental Science, College of Chemistry and Chemical Engineering, Northeast Petroleum University, Daqing 163318, PR China

E-mail: [wangbh@nepu.edu.cn](mailto:wangbh@nepu.edu.cn)

**1. Structure of SDBS molecule**


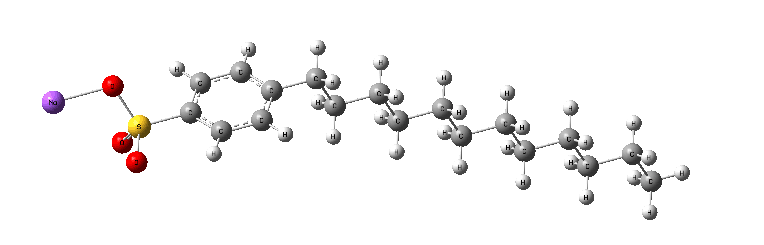


Fig.S1 Structure of SDBS molecule

**2. Thermodynamic parameters at different temperatures**

Table S1 Calculated Gibbs free energy(kJ/mol) and electrochemical potential (V) of full cell reaction at different temperatures using B3LYP/6-31G(d) level of theory

| Temperature/K | 298 | 303 | 323 | 343 | 363 |
| --- | --- | --- | --- | --- | --- |
| △_r_G°_m_ | 1407.27 | 1391.25 | 1329.45 | 1263.97 | 1200.01 |
| E_T_ | 0.1430 | 0.1414 | 0.1351 | 0.1284 | 0.1219 |

Note: Zero-point energies were scaled by 0.9806.

**3. Experimental Apparatus of STEP**


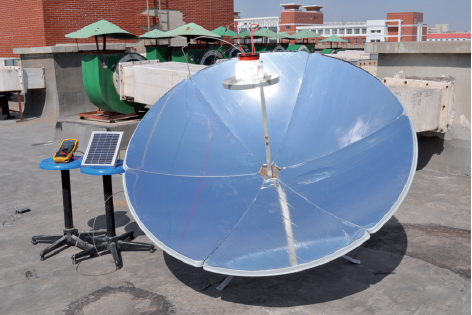


**Synthesis reactor**

**Focus mirror**

**PV module**

**Multimeter**


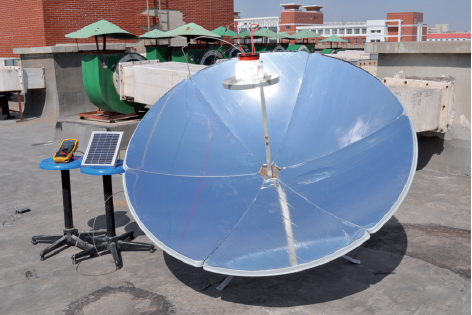


**reactor**

**Focus mirror**

**PV module**

**Multimeter**

Fig. S2. Experimental apparatus picture of the STEP wastewater experiments

If a reflector is axially symmetrical and shaped so its cross-section is a [parabola](http://en.wikipedia.org/wiki/Parabola), it has the property of bringing light that has come from a very distant source such as the sun, so the rays of light are effectively parallel, to a point [focus](http://en.wikipedia.org/wiki/Focus). If the axis of symmetry is aimed at the sun, any object that is located at the focus receives highly concentrated sunlight, and therefore becomes very hot. This is the basis for the use of this kind of solar thermal concentrator.

**Solar thermo-unit.** In the experiments, a paraboloidal solar concentrator (Φ1.5M; Max. Temp. 500℃) was used for focusing sunlight at high magnification. It was equipped with a 2-dimensional tracking reflector control system to move the receiver to intersect this line. Wastewater (or steam) was produced by locating a flow reactor (or reservoir) at the focal point of the solar concentrator for temperatures from RT to 500 ^o^C tuned by the tracking control system. The solar concentrator consists of three main parts. A parabolic reflector serves to concentrate a beam of sunlight more than 1.5 m in diameter to a spot about 5 cm in diameter. The manual control system allows the reflector to be set facing the sun and keeps the reactor at the focal point regardless of the reflector tilt angle. The stand holds the reflector and control system together and allows the reactor to be rotated to follow the sun as it moves across the sky. The amount of heat required (*Q_required_*) and maximum output heat per unit time (*Q_output_*) can be represented as:

*Q_required_ = m· c (t_f_ - t_i_) (1)*

*Q_output_ = H·A (2)*

where, *m* is the, water mass (kg); *c* is the water heat capacity (kJ/kg·^o^C); *t_f_* is the final temperature (^o^C); *t_i_* is the initial temperature (^o^C); *H* is the solar radiation (kJ/m^2^·min), and *A* is the concentrator area (m^2^). The nominal efficiency of the paraboloidal solar concentrator was 65% for heating water. The average daily solar radiation in our city is 48k J/m^2^·min (average day sunlight is 3.5 h per day in Daqing City, Heilongjiang Province). The *Q_output_* of the concentrator in out experiment is 55 kJ/ min, and the *Q_required_* of the reactor is 27.3 kJ from room temperature to 90 ^o^C, so it can far more energy to the reaction.

The constant temperature of the reactor was dependently controlled by tuning the solar radiation intensity, actually, varying the solar effective flux, i.e, effective concentrator area (m^2^) by changing the axis of symmetric face of concentrator to solar.

**Solar Electro-unit.** A polycrystalline silicon solar module (PV cell) equipped with a Li storage battery (5 V, 50 Ah, China) wasused for the solar electronic unit. The cell size is 9.5×6.5 cm^2^, The specifications were tested using an open-circuit voltage of 5 V (V_oc_). The voltage was adjusted for practical use by a voltage divider.

**Solar Reactor.** As shown and described in the above section, an integrated thermo-electrochemical reactor driven by the solar cell, was built for steady-flow tests. The solar STEP-SDBS oxidation reactor was fabricated according to the schematic

anode/(electrolyte + wastewater)/cathode

in a sealed cell (100 mL) capable of being heated by solar radiation. The heating, electrolysis and pump units were solar powered, The paraboloidal solar concentrator was equipped to heat wastewater in the range from RT to 500 ^o^C ( Max.). The solar PV cell supplied the electricity for the oxidation and storage. The thermo-electrolysis energy is extracted from the two units. The energy efficiency of SDBS conversion depends on the level of solar heat inclusion and the coupling of the two unit processes.

**4. Kinetic Equation of SDBS Degradation**

Table S2 kinetic equation of SDBS degradation under different temperature

| Temperature | Kinetic equation | Correlation degree |
| --- | --- | --- |
| 30℃ | y=0.00572x+0.04208 | 0.95482 |
| 50℃ | y=0.00724x+0.07502 | 0.96545 |
| 70℃ | y=0.00996x+0.05455 | 0.98248 |
| 90℃ | y=0.01199x+0.15327 | 0.96654 |

Fig. S3. Fig. 6D with equation for first order plot along with correlation coefficient
